# Visual attention around a hand location localized by proprioceptive information

**DOI:** 10.1093/texcom/tgac005

**Published:** 2022-02-07

**Authors:** Satoshi Shioiri, Takumi Sasada, Ryota Nishikawa

**Affiliations:** 1 Research Institute of Electrical Communication, Tohoku University, Sendai 980-8577, Japan; 2 Graduate School of Information Sciences, Tohoku University, Sendai 980-8579, Japan

**Keywords:** electroencephalogram, hand proximity attention, handedness, steady-state visual evoked potential, visual attention

## Abstract

Facilitation of visual processing has been reported in the space near the hand. To understand the underlying mechanism of hand proximity attention, we conducted experiments that isolated hand-related effects from top–down attention, proprioceptive information from visual information, the position effect from the influence of action, and the distance effect from the peripersonal effect. The flash-lag effect was used as an index of attentional modulation. Because the results showed that the flash-lag effect was smaller at locations near the hand, we concluded that there was a facilitation effect of the visual stimuli around the hand location identified through proprioceptive information. This was confirmed by conventional reaction time measures. We also measured steady-state visual evoked potential (SSVEP) in order to investigate the spatial properties of hand proximity attention and top–down attention. The results showed that SSVEP reflects the effect of top–down attention but not that of hand proximity attention. This suggests that the site of hand proximity attention is at a later stage of visual processing, assuming that SSVEP responds to neural activities at the early stages. The results of left-handers differed from those of right-handers, and this is discussed in relation to handedness variation.

## Introduction

Attention is one of the most important functions of the human brain. In the case of vision, for example, the brain receives a vast amount of input from the retinas each second a person is awake and must decide what to process by selecting the appropriate information. This selection process, known as visual attention, has been widely investigated, and a number of studies have revealed that subjective intention (endogenous or top–down attention) or abrupt changes in stimulation (exogenous or bottom–up attention) changes where attention is focused (Connor et al. 2004; Ogawa and Komatsu 2004; Carrasco 2011), with a certain spatial size as suggested by the spotlight/zoom-lens metaphor (Downing and Picker 1985; Eriksen and St James 1986; Matsubara et al. 2007; Palmer and Moore 2009; Shioiri et al. 2010, 2016). An important aspect of selective attention is the filtration of information that is passed on to later stages of brain processing in which recognition of the complex world filled with various people, objects, and so on is performed (Itti et al. 1998; Luck and Vogel 1998; Carrasco 2011). Selective attention is crucial for action as well as for cognition (Norman and Shallice 1983; Allport 1987) because deciding which body part to move and how to so requires selecting the location of interaction. Despite the importance of selective attention, few studies have investigated the attention mechanisms related to action and/or the body, comparing those related to perception and cognition. Some studies have investigated hand-related attention, considering attentional processes specific to the peripersonal space. The peripersonal space is a concept of internal representation of the world, which describes the environment close to the body (Rizzolatti, Gentilucci, et al. 1987a; Rizzolatti, Riggio, et al. 1987b). Reed, Grubb et al. (2006) reported a type of visual attention related to body parts and showed prioritized attention for visual stimuli close to one’s hand, a finding that was supported by subsequent studies (Abrams et al. 2008; Tseng and Bridgeman 2011; Perry and Fallah 2017; Reed et al. 2018). Visual attention tends to move to the location of the hand, perhaps to improve visual processing required near the hand and to execute actions. Other studies investigated the attention paid to the goal of hand movement, which might result in improved visual processing for the action after a hand movement (Deubel et al. 1998; Rowe et al. 2002; Eimer et al. 2005; Baldauf et al. 2006; Baldauf and Deubel, 2008, 2009, 2010; Gutteling et al. 2011, 2015; Mason et al. 2015; Miura et al. 2017; Hanning et al. 2018).

The hand proximity effect, or attention, has been identified through a variety of conditions, often measuring the reaction time or sensitivity to detect a target (Reed, Stone, et al. 2006b; Abrams et al. 2008; Tseng and Bridgeman 2011; Gozli et al. 2012; Reed et al. 2018), but other tasks have also been used, including visual search (Abrams et al. 2008), figure–ground assignment (Cosman and Vecera 2010), and memory-related tasks (Tseng and Bridgeman 2011). The basic finding is the facilitation of visual processing (e.g. shortening of reaction time) when one’s hand is near the stimulus display compared with when it is on one’s lap, which has been widely reported, while another aspect of hand proximity is the slowing disengagement of visual attention (Vatterott and Vecera 2013; Bush and Vecera 2014). This suggests that there might be one or more specialized processes that facilitate visual processing in the peripersonal space, particularly the space around the hands. However, the empirical results have not elucidated the underlying mechanism, for which there are at least 2 possibilities. One is facilitation by attention via the recruitment of bimodal visual–tactile neurons representing the hands and the space near the hands. The other is preferential processing of stimuli near the hands by the action-oriented magnocellular visual pathway (Taylor et al. 2015; Caplette et al. 2017). These 2 interpretations differ in terms of the sites of interaction between vision and hand location, although it is possible that both of the mechanisms contribute to hand proximity attention (Tseng et al. 2012; Taylor et al. 2015; Tseng and Lo 2020). To investigate the site of the interaction under various conditions, it is critical to first investigate the effect while controlling for factors that might potentially contribute to the effect. Accordingly, in this study, we attempted to investigate the underlying mechanism of hand proximity attention by considering the following 5 factors. We focus on the 2 possible underlying mechanisms of hand proximity attention in Section 4.

The first factor is the effect of the top–down attention. A simple explanation of the facilitation of visual processing around one’s hand is top–down attention. Hand proximity attention may be a bias of top–down attention to the location of one’s hand, which facilitates visual processing near the hand (Qian et al. 2012; Garza et al. 2013). Garza et al. (2013) found a biasing effect of the hand on target detection when instructions emphasized the location of the hand near targets but not when the location of the other hand, which was used to make responses, was emphasized. Qian et al. (2012) also found a biasing effect of the hand only when the stimuli near the hand were relevant to the task. These studies suggest that the presence of the hand itself may imply a task-related context, biasing participants’ expectations as to where important stimuli might occur. However, hand proximity attention was reported on a stimulus in the central visual field where top–down attention was likely focused because there was no other task-related stimulus at other locations (Garza et al. 2018). That is, visual performance is influenced by the location of one’s hands even when there is no reason to shift attention to them in order to perform a given task. However, no previous study intentionally controlled top–down attention between the 2 locations at an equivalent distance from the point of fixation, except for preliminary reports from the present study published in abstract form (Shioiri et al. 2010; Nishikawa et al. 2014). We designed experimental conditions to isolate hand proximity attention from top–down attention, instructing participants to pay attention to one of the two locations with a hand at either the same or a different location. The difference between the 2 conditions can be attributed to hand proximity attention because top–down attention was oriented to the same point between the two.

The second factor is the effect of visual information. Many experiments compared visual performance with a hand near the stimulus display versus on one’s lap, such that either(both) visual or(and) proprioceptive information contributed to visual facilitation near the hand. A few experiments showed enhanced facilitation of visual stimulus even when the hand was not seen, although the effect was weaker than with visual information (Garza et al. 2013, 2018; Reed et al. 2013; Brown et al. 2015). Moreover, one study showed facilitation of tactile processing on a hand preparing to move (Forster and Eimer 2007), and another study demonstrated the influence of hand position on visual sensitivity in a patient with severe left hemianopsia; detection of targets in his “blind” field improved when his left arm was placed near the target stimuli (Schendel and Robertson 2004). These results suggest that both visual and proprioceptive information contribute to hand proximity attention. To investigate the underlying mechanism, we designed experimental conditions to isolate proprioceptive information from visual information. The visual stimulus was presented at the exact location of a hand under the condition, where the hand was not seen because it was covered by a mirror that reflected light from a display, causing the visual stimulus to appear at the palm of the invisible hand.

The third factor is action to respond. There is a report of hand proximity attention for moving hands (Adam et al. 2012). Reaction time measurements are typically performed to investigate the effect of the visual attention and are also used for hand proximity attention. When measuring reaction time, participants are likely to pay attention to their hand. Therefore, to prevent any influence on the measurements, we should avoid this factor. In this study, we used the flash-lag effect (FLE) to avoid prompting responses during measurement trials. The FLE is a phenomenon that occurs when a flash aligned with a moving object is perceived to lag behind that object (Whitney and Murakami 1998; Krekelberg et al. 2000; Murakami 2001a, 2001b; Hogendoorn 2020). Several theories have been proposed to explain the FLE, including extrapolation of motion trajectory (Nijhawan 1994), differential latencies (Whitney and Murakami 1998; Murakami 2001a, 2001b), motion bias (Eagleman and Sejnowski 2000), attention (Baldo and Klein 1995), and so on (Nijhawan 2008). It has been reported that, among other factors, attention contributes to the FLE, which is known to be reduced by attention, and that the FLE can be used to measure the degree of attention and has been confirmed to show similar results with other measures for visual attention (Baldo and Klein 1995; Baldo and Namba 2002; Namba and Baldo 2004; Kashiwase et al. 2012; Hogendoorn 2020). Given that the effect size can be indicated after the stimulus termination, no influence of the action is expected on the measure.

The fourth factor is the effect of the peripersonal space. As mentioned above, the peripersonal space is an internal representation of the world that describes the environment in close proximity to one’s body (Rizzolatti, Gentilucci, et al. 1987a; Rizzolatti, Riggio, et al. 1987b), and hand proximity attention is considered to facilitate visual processing in the peripersonal space. However, the effect may depend on the distance between the hand and the stimulus, even within the peripersonal space. That is, it is not clear whether hand proximity attention is an effect specific to the entire peripersonal space or only around the hands. Previous studies have usually compared the conditions with a hand near the stimulus display versus that with the hand far from the display, such as on one’s lap. If the visual information is enhanced to realize better manual control by hand proximity attention, facilitation is expected to be localized around the hand in action, rather than spreading over the space near the body. In contrast, if hand proximity attention is the result of general visual processing in the peripersonal space, we expect no difference in visual processing between areas near and far from the hand within the peripersonal space. Some previous studies have reported a similar effect for stimuli presented near and far from the hand as long as the stimuli were on the display near the hand. However, no study has focused on a direct comparison between the locations from the hand near the display.

Finally, we also investigated the effect of handedness. If hand proximity attention functions to help hand actions, the dominant hand is expected to have a larger effect. However, there may not be much asymmetry between the dominant and nondominant hands if the underlying mechanism of hand proximity attention is at the very early stage of visual processing, where the difference between them cannot be detected.

We also attempted to estimate the spatial profile of attention modulation by taking electroencephalograms (EEGs). Several studies have reported the modulation of brain activities corresponding to hand proximity attention (Abrams et al. 2008; Reed et al. 2017, 2018). Our interest is in the spatial range of attention, which is suggested to differ according to the process. There is a suggestion for narrow tuning at a later stage and for broader tuning at an earlier stage of visual processing (Shioiri et al. 2016). To estimate the spatial profile of hand proximity attention, we applied a technique called steady-state visual evoked potential (SSVEP) as used in previous studies of spatial/temporal attention (Muller, Picton, et al. 1998a; Muller, Teder-Salejarvi, et al. 1998b; Kim et al. 2007; Andersen and Muller 2010; Andersen et al. 2011; Kashiwase et al. 2012, 2013; Dmochowski et al.^2015^; Shioiri et al. 2016; Mora-Cortes et al. 2018), feature-based or object-based attention (Andersen et al. 2015; Kuriki et al. 2015; Adamian et al. 2020), and attention for moving objects (Stormer et al. 2013; Stormer et al. 2014). The spatial property of hand proximity attention may be similar to that of high-level visual processing reported in a previous study (Shioiri et al. 2016). As for high-level visual processing, attention may be focused locally at the location of the hand, similar to the attention effect at hand-movement goals (Deubel et al. 1998; Baldauf et al. 2006; Baldauf and Deubel 2008, 2010).

Furthermore, we compared the effect of hand proximity attention between left- and right-handers. Although the cause of variation in handedness (only about 10% of people are left-handed) remains unclear despite decades of research (McManus 2019), handedness might have some relationship with hand proximity attention. For example, stronger hand proximity attention was found around the right hand in right- handers (Tseng and Bridgeman 2011), and a weaker effect was found for left-handers compared with right-handers (Le Bigot and Grosjean 2012). Therefore, in this study, we investigated the possible differences in hand proximity attention between left- and right-handers while controlling for the factors described above.

## Experiment 1: FLE and SSVEP measurements

**Fig. 1:**
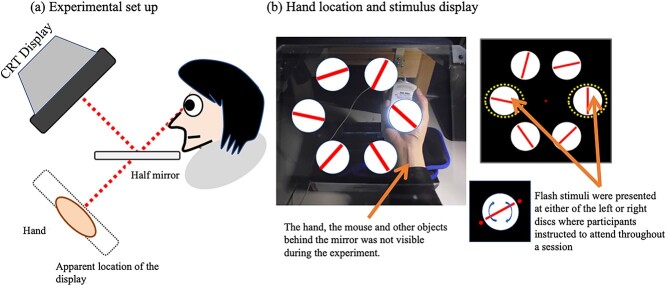
a) Experimental setup. The display was observed through a mirror so that one of the discs was perceived as being on the palm of the hand, which was not visible. b) Left: Visual stimuli and the hand location (not visible during the experiment). Right: Flashes are presented at either the left or right disc and the perceived location of the bar relative to the flashes was reproduced at the end of the trial.

We measured attentional modulation on a visual task under conditions in which the hand was either near or far, using a visual display with 6 discs (Fig. 1b). Either the left or the right hand was placed at the location of either the left or right disc and the other hand was on the lap. The participants looked at visual stimuli through a mirror at the location of their hand, which was hidden by the mirror (Fig. 1a). We used FLE to measure the attention effect without any hand movement during the stimulus presentation. Given that FLE is smaller at the location where attention is focused, attentional modulation can be estimated by measuring FLE (Shioiri et al. 2010).

EEG signals were measured to investigate the spatial profile of attentional modulation related to hand proximity attention as well as that related to top–down attention by using SSVEP analysis. To that end, 6 discs arranged in a circle at the same distance from the central fixation point flickered at different temporal frequencies (frequency tagging). To examine how the distance from the location of attentional focus influences the neural response to each flickering stimulus, we extracted each temporal frequency component from the EEG signal. The change in amplitude of each frequency component was obtained as the measure of attentional modulation: the higher the amplitude, the larger the effect of attention.

### Methods

We conducted an experiment under the condition where top–down attention was fixed at a location. Only the visual stimulus on the display was visible; the hand and other objects were not visible during the trial. No response was required during stimulus presentation. To control for top–down attention, the participant was told the location of the task-related stimulus before each session and they were asked to focus on that location throughout the session. There were 4 conditions related to the hand location (left hand at the left side of fixation, left hand at the right side of fixation, right hand at the left side of fixation, and right hand at the right side of fixation), and the condition remained the same throughout a given session. There were 8 combinations of the 4 hand conditions and 2 stimulus locations (top–down attention on the left or right stimulus), and 2 sessions were performed by each participant for each of the 8 conditions. We measured the FLE at the location of attention by asking the participant to reproduce the apparent location of the moving bar relative to the flash. This setting was performed after each trial so that no action was necessary during the stimulus presentation.

### Participants

Participants were 20 right-handers and 18 left-handers with normal or corrected-to-normal visual acuity. After excluding those with frequent eye movements during the trials, 16 participants in each group [13 men and 3 women in the right-handed group, average age 22.4 years (range 22–25 years); 7 men and 9 women in the left-handed group, average age 22.8 years (range 21–24 years)] were included in the analysis. All participants provided written informed consent before the experiment. The experiments were approved by the Ethics Committee of the Research Institute of Electrical Communication, Tohoku University and were carried out in accordance with approved guidelines.

Handedness was scored using the Japanese version (Ocklenburg et al. 2010) of the Flanders handedness test (Nicholls et al. 2013). The test contains 10 questions asking which hand(s) the person uses for various actions, including writing, eating with a spoon, brushing one’s teeth, striking a match, drawing a picture, erasing a pencil mark, holding the needle when sewing, holding the knife when buttering bread, holding a hammer, and holding the peeler when peeling an apple. Answering “right hand” gives a score of 1, “left hand” a score of −1, and “both” a score of 0, for a total score ranging from −10 to 10. The scores of left-handed participants ranged from −10 to 8, whereas those of the right-handed participants were all 10 (i.e. they use their right hand in all 10 situations). The participants were assigned to the left- and right-handed grouped based on their self-evaluations because there seems to be no agreement on the method for classifying handedness (Doyen and Carlier 2002; Busch et al. 2010). It may better to classify people as right-handers and non-right-handers, but we used the term left-handers, following the participants’ own self-evaluation. We used the handedness score to analyze correlations with the effect of hand proximity attention.

### Stimuli

The experimental setup is shown in Fig. 1a. Participants looked at visual stimuli on a cathode ray tube (CRT) display through a half mirror (20 × 20 cm) without seeing the hand hidden by the mirror when the light under the mirror was off. The spatial resolution of the display was 800 × 600 pixels and the frame rate was 140 Hz. Either the left or right hand was placed at the location of either the left or right disc. The hand placed at the location of the disc held a mouse, which was used to report the FLE after each trial. The stimulus configuration is shown in Fig. 1b. To analyze SSVEP, 6 discs flickered at the following temporal frequencies: 8.2, 9.4, 10.7, 12.3, 14.0, and 15.6 Hz. These frequencies were selected to be clearly visible with the luminance contrast of the flicker used (97.7%, the maximum contrast available) and not to have the same harmonics within the frequency range of interest. The average luminance of the discs was 75.8 cd/m^2^ and the diameter was 7.5°. The distance of each flickering disc from the fixation point was 10.0° from center to center. A red bar was presented on each disc and 2 small red discs were presented briefly (flash stimulus) near the ends of the bar. The luminance and color coordinates of the moving red bar and the red flash stimulus were 35.5 cd/m^2^ and 0.610, 0.340, respectively. The scale of the bar was 0.6° in width and 7.0° in height, and the diameter of the red flash stimulus was 1.1°. The red bar rotated about the center of the disc at a speed of 0.97 rps (350° rotation per second). The bars on the 6 discs rotated in the same direction in 1 session and changed between the 2 sessions of the same condition so that sessions were carried out under each condition with both clockwise and counterclockwise rotations. The initial angle of the bar was selected randomly for each of 6 discs from trial to trial. Small red discs flashed at either the left or right disc during bar rotation in accordance with the information given before the session. The flash presentation location was 4.6° from the center of the disc. The location of the bar was changed every 2 frames (14.2 ms) and the duration of the flash was also 2 frames.

### Procedure

**Fig. 2:**
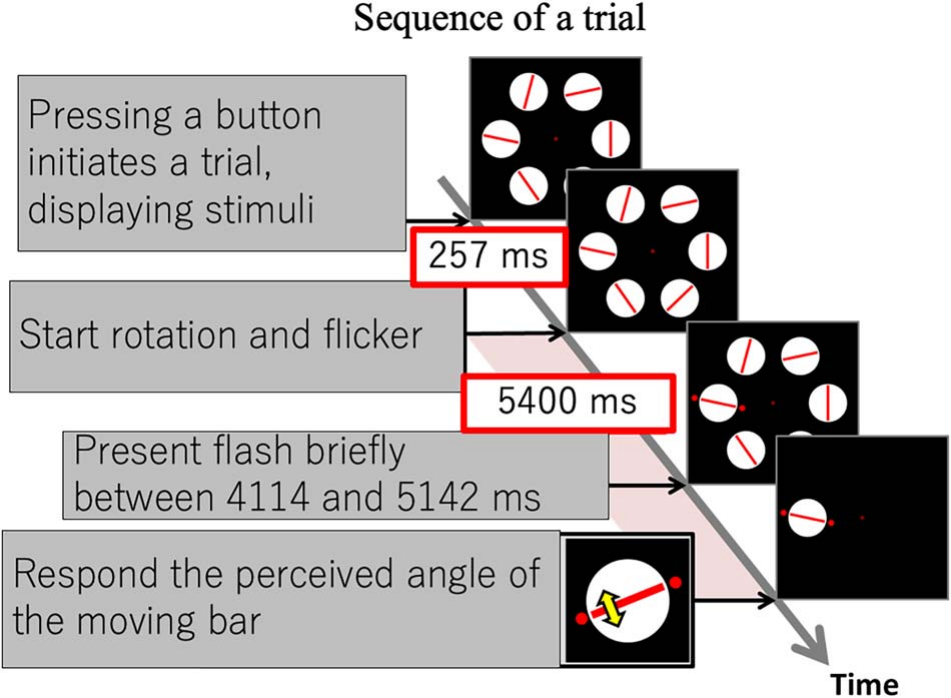
Trial sequence. Stationary discs and red bars were presented when the participant pressed a button of the mouse they were holding. The bars started rotating and the discs started flickering at 257 ms after the stimulus display presentation and lasted for 5,400 ms. Between 4,114 and 5,142 ms after the start of bar rotation and disc flicker, small red discs were presented at a point close to the rotating bar at the focus of attention. At the end of trial, the response display was presented and the participant reproduced the relationship between the bar and the flashes.

Before starting a session, the hand location was confirmed to be at either the left or right disc, and the participant was looking at the overlapped images of the visual stimulus and their hand through the half mirror, with the hand lit under the mirror (Fig. 1b). The participant was instructed to keep their other hand on their lap. The trial was initiated when the participant clicked the button on the mouse (Fig. 2). At 257 ms after the mouse click, red bars started rotating and the discs started flickering. At the same time, the participant focused their attention on the disc subjected to the flash presentation. The perceived difference between the flashes and the moving bar was reproduced in the response display presented after each trial by moving the bar relative to the stationary red discs. The red discs flashed with randomized delays of between 4,114 and 5,142 ms after the start of the trial. The stimulus image was replaced by the response display when 5,400 ms had elapsed. The response display consisted of 1 white disc with a red bar and 2 red discs at the location of the flashes and the fixation point. In the response display, the participant used 2 mouse buttons to control the bar angle in order to reproduce the perceived difference between the flashes and the bar. One click corresponded to 0.25° rotation of the bar, with the left button rotating the bar clockwise and the right button rotating it counterclockwise. The participant clicked the center button when they were satisfied with the location of the bar angle relative to the red discs on the display. There were 8 different conditions, and 2 sessions were performed for each condition for each participant. Each session consisted of 48 trials. The bar and flashes were aligned in 42 trials, and the angle between them was randomly set at between 10° and 30° in 8 dummy trials, which were not analyzed. Six different combinations of flicker frequencies and locations were used to minimize any interaction between location and frequency, and these combinations were used randomly within a given session (arranged from low to high in the counterclockwise direction, starting at either of the 6 randomly chosen locations for each trial). All 6 frequency arrangements were used for each of the 8 conditions for a total of 48 trial combinations per session. The condition order was fixed for half of the participants as left-hand/left-side, left-hand/right-side, right-hand/left-side, and right-hand/right-side for each of the left-flash and right-flash conditions, with the left-flash condition presented first. Counterclockwise bar rotation was used in the first session, and clockwise bar rotation was used in the second session for half of the participants; this order was reversed for the other half of the participants.

### E‌EG recording

Following the procedure used in previous studies (Kashiwase et al. 2012; Kuriki et al. 2015; Shioiri et al. 2016), we recorded brain electrical activity from 19 scalp electrodes mounted on an elastic cap connected to an EEG recording system (Neurofax EEG-9100; Nihon Koden, Tokyo, Japan). The electrode arrangement was based on the International 10–20 System, with reference electrodes placed on both ear lobes. EEG signals were recorded using a 120-Hz high-pass filter, digitized at 1,000 Hz and stored for off-line analysis. All electrode impedances were confirmed to be below 5 kΩ before each session. To ensure that any SSVEP modulation observed in the experiment was attributable to an attentional effect, participants were asked to fixate on the center of the display and to try not to blink during each 6-s trial. We also recorded horizontal eye movements based on the signals from 2 additional electrodes placed 1 cm above and below the outer canthus of the left and right eyes. We excluded trials with electrooculogram (EOG) deflections of more than ±50 V, which corresponds roughly to a 5° eye shift. This also excluded trials with eye blinks. Data from 4 of 20 right-handers and 2 of 18 left-handers were excluded from the analysis because of high rates of trial rejection (>30%).

### S‌SVEP analysis

For the frequency analyses, we selected data from between 3,114 and 4,614 ms after stimulus onset in order to obtain the attention stage right before the flash presentations. Given that the task-relevant stimuli were presented between 4,114 and 5,114 ms after the stimulus presentation, attention may not be oriented to the flash location until right before the flash presentation. We included the data from 4,114 to 4,614, assuming an interval of 500 ms or more between the stimulus presentation and the expected time required for retinal stimulation to have an effect on attention (Kashiwase et al. 2012). The results were similar when the data between 3,114 and 4,114 ms were used.

We focused on the channels of 3 electrodes (O1, O2, and Pz) based on the finding that these channels showed the largest attentional modulation (see Fig. 7). The data from the 3 channels were averaged for the analysis. To analyze the frequency characteristics, EEG data for each trial were transformed for the frequency domain by fast Fourier transform using a 20% tapered cosine window, which yielded a frequency resolution of 0.5 Hz. Data were normalized separately for each temporal frequency in order to eliminate the influence of differences in sensitivity to different temporal frequencies. After calculating a *Z*-score to normalize the data for each frequency, the change in amplitude as a function of the distance between the corresponding disc and the hand or the flash location was attributed to a purely attentional effect. We averaged the amplitude score of all temporal frequencies and then calculated the *Z*-score again for each participant from the averaged data over different frequencies in order to normalize the individual differences in attentional modulation.

### Results

**Fig. 3:**
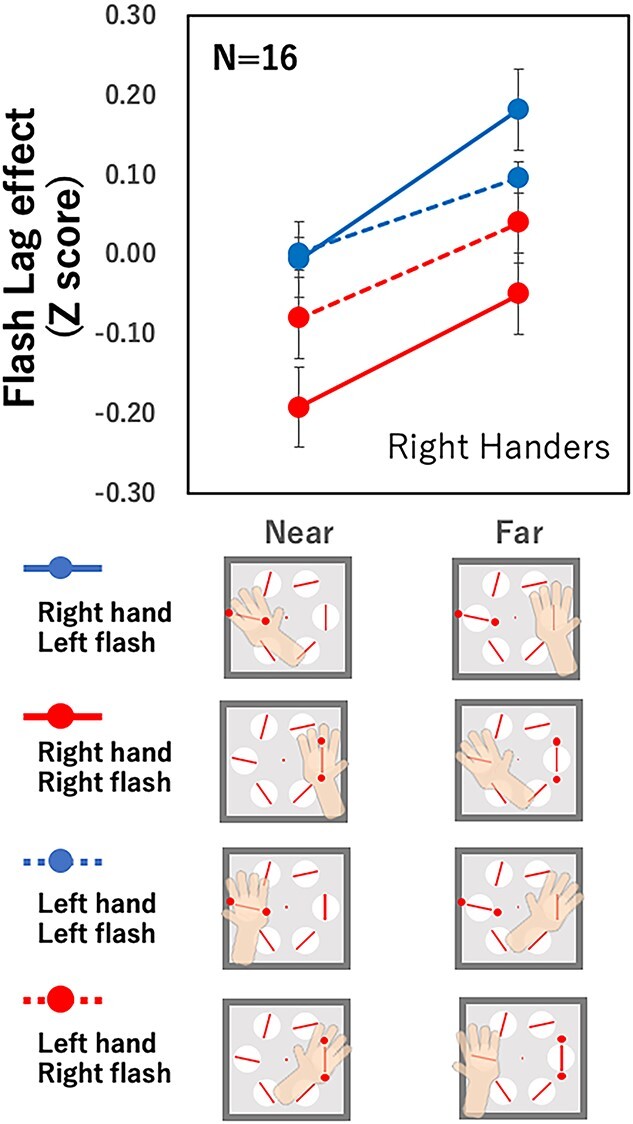
FLE for right-handers. Solid and dashed lines show the right and left hand conditions, and white and black symbols show the right and left flash conditions, respectively. The vertical axis indicates the FLE averaged over 16 participants after normalizing as *Z*-scores among the 8 conditions for each participant.

Figure 3 compares the size of the FLE between the near- and far-hand conditions for right-handers for each of the 4 conditions (left hand/left side, left hand/right side, right hand/left side, and right hand/right side). A smaller FLE was found when the hand was positioned near the flash than when it was positioned far from the flash under all 4 conditions. Three-way analysis of variance (ANOVA) for the hand used (right/left), hand location (right/left), and distance between the flash and the hand (near/far) showed significant main effects of the distance between the flash, the hand used, and the flash position, as well as a significant interaction between the hand used and the flash position [*F*(1,15) = 11.38, *P* = 0.004, η_p_^2^ = 0.43; *F*(1,15) = 7.92, *P* = 0.013, η_p_^2^ = 0.35] and interaction between hand and flash location [*F*(1,15) = 9.84, *P* = 0.007, η_p_^2^ = 0.40]. No other significant main effect or interaction was found (*P*s > 0.1). The smaller FLE at the location near the hand compared with that far from the hand indicates that there was an attention effect depending on the hand location, even when the hand location is not relevant to the task. The participants’ task was to pay attention to the location, ignoring the hand position. We also found that attentional modulation was larger for the flash position to the right of the fixation point compared with that to the left. Because a clear difference between the left and right flash positions can be seen for the right hand in Fig. 3 and because there is a significant interaction between the hand and flash location, it is likely that the effect of flash location is more prominent for the right hand than for the left. Right flashes attracted more attention when the right hand was used, independent of the hand position.

**Fig. 4:**
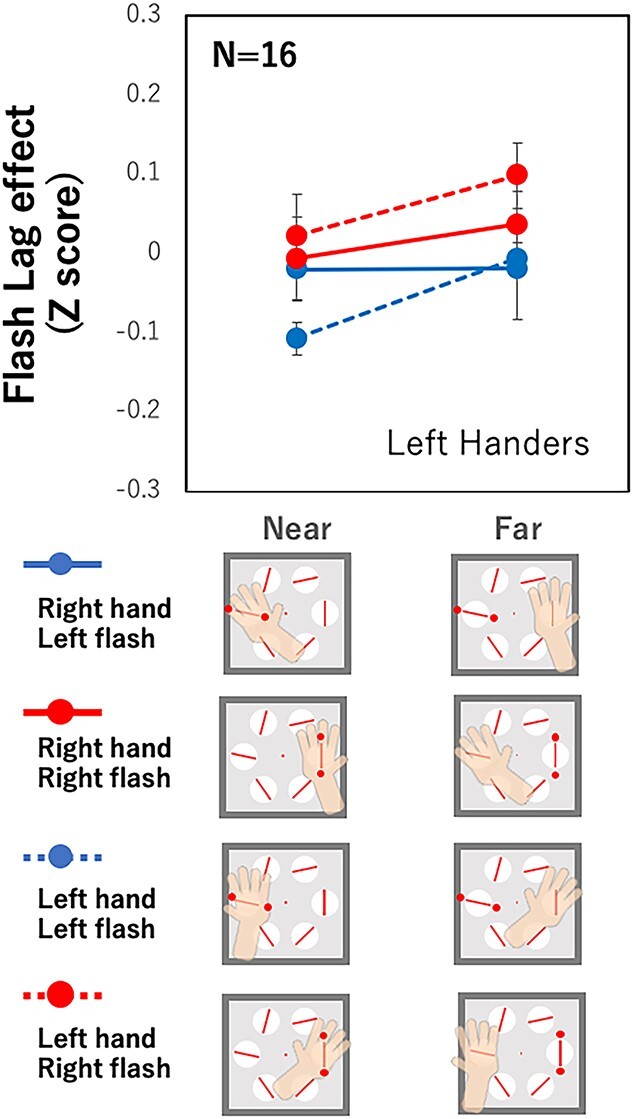
Flash lag effect for left-handers. The notations are the same as in Fig. 3.

Figure 4 shows the results of the left-handers. We applied the same 3-way ANOVA as that applied to the right-handers. The results showed no significant main effect or interaction (*P*s > 0.1), although the FLE tended to be smaller for the near-hand condition compared with the far-hand condition. The results suggest that hand proximity attention, if present, is weaker in the left-handed group than in the right-handed group. However, it should be noted that these are the group results. We used Welch’s *t*-test to check for differences between the near and far conditions in each participant, using FLE data from all trials; we found that 6 of the 16 left-handers showed a significant effect of hand proximity attention. This number is slightly less than that of the right-handers, half of whom (8 of 16) showed a significant effect of hand proximity attention. We also found that 3 of the 16 left-handers showed a larger FLE when their hand was near rather than far, which was the opposite effect of hand proximity attention. This was not expected and we do not know how to interpret this result.

**Fig. 5:**
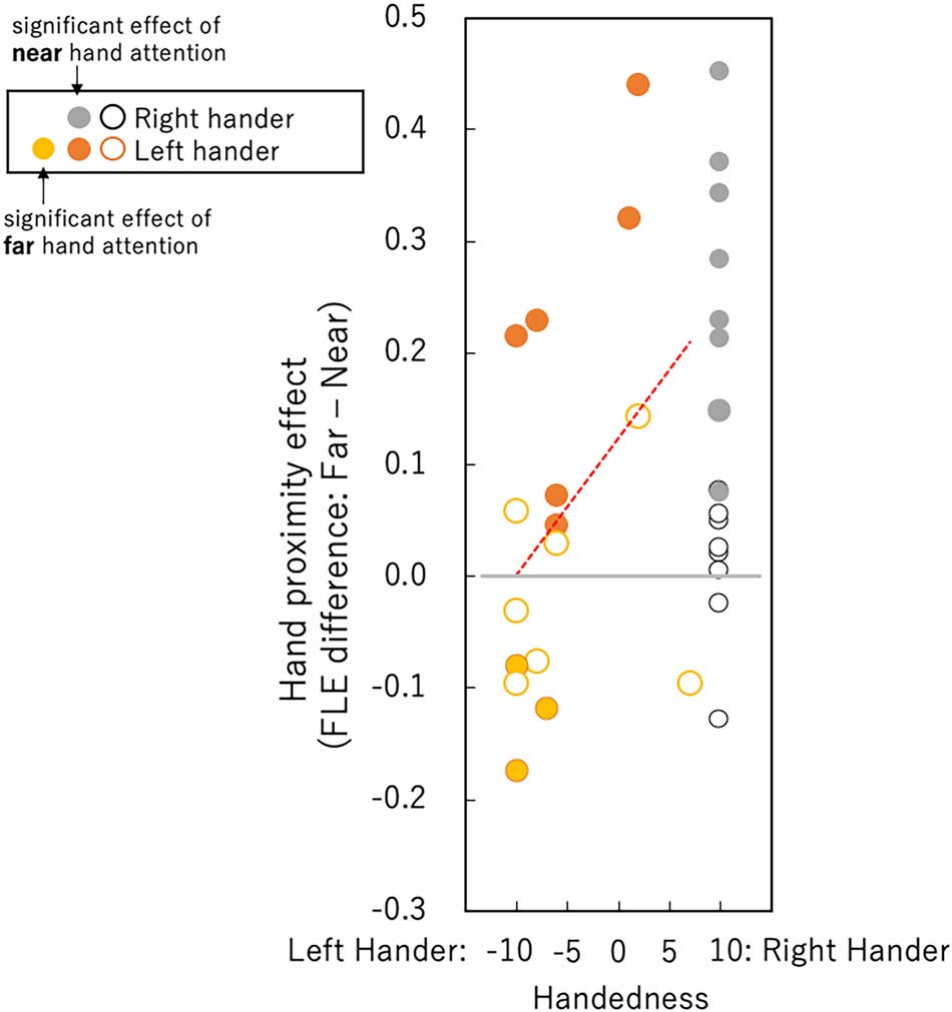
Hand proximity effect, calculated by subtracting the FLE in the near condition from that in the far condition, as a function of handedness score. Small circles represent the results of right-handers, large circles represent the results of left-handers, the latter of which are defined as people who use their left hand dominantly in at least 1 of the 10 situations in the handedness evaluation questionnaire and who describe themselves as left-handed. Closed circles indicate the results of participants who showed a statistical difference between the near and far conditions, and open circles represent the results of those who did not. Closed black circles indicate a hand proximity effect, whereas gray circles indicate the opposite effect, in which a smaller FLE was observed in the far condition than in the near condition.

Figure 5 shows the effect of hand proximity attention as a function of handedness score. The closed circles indicate participants who showed a significant difference between the near and far conditions. There is a marginal significant correlation between the effect of hand proximity attention and handedness [*r* = 0.34, *t*(30) = 1.97, *P* = 0.058]. This effect tends to be larger with larger handedness scores, which appears to show that hand proximity attention is related to the usage of the right hand. Indeed, the average effect size of hand proximity attention was around zero for left-handers with a handedness score of −10. However, 4 of the 12 left-handers with handedness scores lower than 0, including 1 of 6 with a score of −10, showed a significant effect of hand proximity attention. This indicates that the group average does not represent individuals. Because a considerable number of left-handers showed a significant effect of hand proximity attention and also because previous studies reported hand proximity attention for left-handers (Le Bigot and Grosjean 2012; Colman et al. 2017), we cannot conclude that the left-handers in the present did not show an effect of hand proximity attention, even though the difference in average FLEs between the near- and far-hand conditions were not statistically significant.

There was another interesting difference between the left- and right-handers in terms of differences in processing stimuli between the left and right visual fields. The FLE was smaller with the right flash for the right-handers [*F*(1,15) = 7.92, *P* = 0.013,η_p_^2^ = 0.35 for the main effect of flash location] and was smaller with the left flash for the left-handers, but this was not statistically significant. In other words, the FLE was smaller when the flash was presented at the location of the dominant hand, and so right-handers showed improved facilitation with the right-side flash and left-handers showed improved facilitation with the left-side flash. Also, the difference tended to be larger for the right hand of the right-handers [*F*(1,15) = 9.84, *P* = 0.007, η_p_^2^ = 0.40 for the interaction between hand and flash location] and larger for the left hand of the left-handers, but this was not statistically significant. A similar effect of hand and stimulus location has been reported (Lloyd et al. 2010). The present results suggest that the underlying mechanism of the visual field effect is symmetrical for left- and right-handers, with some stronger attentional modulations for right-handers. These effects of handedness on the different attention modulations of the left and right visual fields are possibly related to the underlying mechanism of handedness, which remains to be elucidated (Badzakova-Trajkov, Haberling, and Corballis 2010a; McManus 2019). The present results may provide new clues for solving this mystery, but that is beyond the scope of this report.

**Fig. 6:**
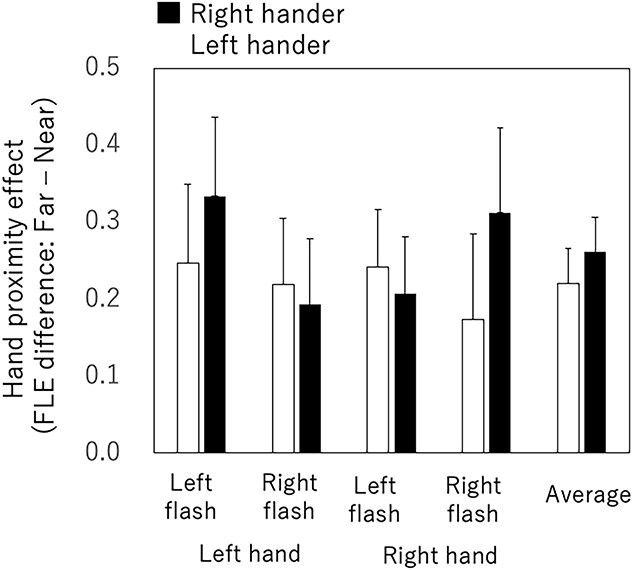
Hand proximity effect for the 4 combinations of the hand used and the flash location. White and black bars represent the left- and right-handers, respectively. Error bars represent the standard error of mean.

We found that 14 of the 32 participants showed a statistically significant effect of hand proximity attention from the FLE measures. Here, we analyzed hand proximity attention by using only the data from participants who showed a statistically significant effect in order to clarify the difference in hand proximity attention among different conditions. Figure 6 shows the effect of hand proximity attention for the 4 combinations of hand and flash locations with the averages for the left- and right-handers. Three-way ANOVA (handedness × hand × flash position) showed no significant main effect or interaction. The effects of hand proximity attention were similar for the left- and right-handers in terms of behavioral results.

**Fig. 7:**
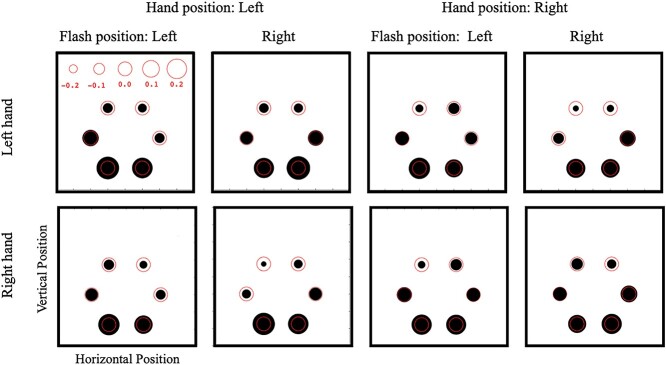
Spatial distributions of SSVEP amplitude for the right-handers. The 6 discs in each panel represent the 6 discs on the stimulus display, and the size of the discs indicate the SSVEP amplitude. The relationship between disc size and *Z*-score are shown at the top of the top left panel. Each panel shows the results of each of 8 conditions: combinations of the hand used, hand position, and flash location.

The purpose of the SSVEP measures was to investigate the spatial spread of visual attention around the hand. We used data from electrodes with the largest 3 SSVEP amplitudes (i.e. O1, O2, and Pz) for further analyses. Figure 7 shows the relative amplitude of SSVEP signals for each location of the 6 flickering discs for the right-handers. The size of the black discs in each panel expresses the *Z*-scores averaged over all participants. The relationship between the score and size is shown at the top of the top left panel, and the red circle plotted at the black disc represents a *Z*-score of zero. Four-way ANOVA (hand × hand position × flash position × disc location) showed a significant main effect of location [*F*(5, 75) = 13.01, *P* < 0.001, η_p_^2^ = 0.46] and significant interactions between location and flash position [*F*(5, 75) = 3.95, *P* = 0.003, η_p_^2^ = 0.41], without any other significant effect or interaction. The results of the left-handers were similar for location effects. A similar result for location effect was found for the left-handers (not shown). The same 4-way ANOVA showed a significant main effect of location [*F*(5, 75) = 5.58, *P* < 0.001, η_p_^2^ = 0.27] and significant interactions between location and flash position [*F*(5, 75) = 3.47, *P* = 0.007, η_p_^2^ = 0.19], without any other significant effect or interaction. Figure 7 shows larger EEG responses to the stimulation at lower visual fields. Asymmetry in visual responses between the upper and lower visual fields has been called the “lower field advantage,” manifested as faster behavioral responses, greater sensitivity, and a faster and larger evoked visual response to lower visual field stimuli (Fioretto et al. 1995; Hagler 2014). Because the location effect was not relevant to our present aims, we analyzed SSVEP amplitude, eliminating the location effect as described below.

**Fig. 8:**
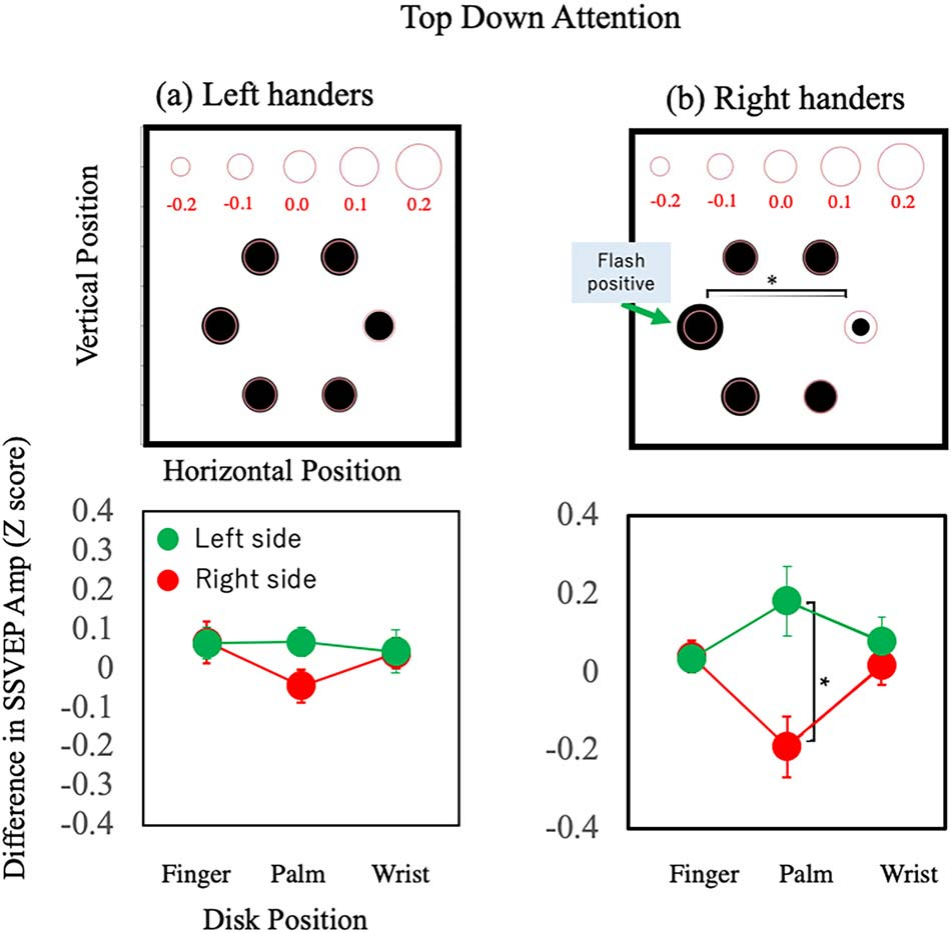
Estimation of the top–down attention effect. The difference in SSVEP amplitude between the results under left-flash and right-flash conditions, with other factors (the hand used and the hand position) being the same. The positive values mean the amplitude is larger when the dots flashed at the left because the values under the right-flash condition were subtracted from those under the left-flash condition. The top panels show the spatial distributions and the bottom panels show the effect of the location relative to the hand; finger, palm, and wrist correspond to the top 2 discs, the middle 2 (left and right) discs, and bottom 2 discs, respectively, in the right-handers. The green discs indicate the results of the left side of the display and the red discs indicate the results of the right side of the display. a) Results of the left-handers. b) Results of the right-handers.

The interaction between flash and flicker location can be attributed to the effect of top–down attention. We expected to have a larger amplitude to the location of the flash where the top–down attention was oriented. We compared the SSVEP amplitude measured when the top–down attention was at the left and right disc (the flashes were presented at the left or right disc), subtracting the latter from the former. The result shows the facilitation effect of top–down attention because the 2 conditions differ only by the location of attention, keeping the other factors (i.e. the hand and its positions) the same. Figure 8 shows the subtraction results for the left-handers (a) and right-handers (b). To summarize the conditions with different flash locations, the results in the right flash conditions were flipped horizontally so that the flash locations were at the left for all conditions. That is, Fig. 8 shows the top–down attention effect averaged over different conditions so that the attention was nominally focused on the left disc. The top 2 panels show the SSVEP amplitudes at each location and the bottom 2 panels compare the results between the attended and unattended sides for each location relative to the hands. The left or right location was labeled as “palm,” the top 2 locations as “finger,” and the bottom 2 as “wrist” and the difference between the left and right sides was tested with a *t*-test for each. For the right-handers, a significant difference was found for palm pair [*t*(15) = 2.5, *P* = 0.03, Cohen’s *d* = 1.12], with no significant effect for other pairs (*P*s > 0.1). The larger SSVEP amplitude at the location of the flash stimuli indicates that top–down attention is oriented to the task-relevant location. The lack of a clear difference between the flash side and the other side of the fixation for the fingers and wrists suggest that the spatial tuning for top–down attention is relatively narrow. There was no significant effect of top–down attention for the left-handers, in contrast with the right-handers.

**Fig. 9:**
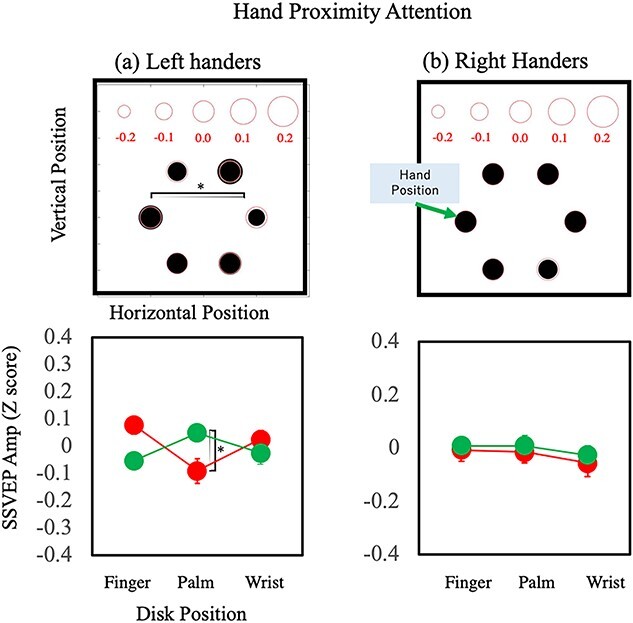
Estimation of the hand proximity attention effect. The difference in SSVEP amplitude between the results under the near- and far-hand conditions, with the other factors (the hand used and the flash location) being the same. The positive values mean the amplitude is larger when the hand is near rather than far from the flash because the values under the near-hand condition were subtracted from those in the far-hand condition. Although the near-hand condition can be at the left or right, we averaged the results of the 2 cases after flipping the results from the right near-hand conditions horizontally. Other notations are the same as in Fig. 8.

To extract the effect of hand proximity attention, we next subtracted the SSVEP amplitude measured when the hand was near the flash from that measured when it was far. This calculation extracts the effect of hand location relative to the flash and removes the effects of absolute locations, that is the hand locations and flash locations for each hand. Figure 9 shows the effect of hand proximity attention for the left- and right-handers. To summarize the conditions with different hand locations, the results under the conditions with the hand at the right were flipped horizontally so that the hand locations were at the left for all conditions. A significant difference was found for the palm pair of the left-handers [*t*(15) = 2.4, *P* = 0.03, Cohen’s *d* = 0.93], with no significant effect for other pairs (*P*s > 0.1) except one: The *t*-test for the finger pair of the left-handers showed a statistical difference between the 2 sides [*t*(15) = 2.6, *P* = 0.02, Cohen’s *d* = 1.03] and the amplitude was larger when the hand was far. Despite the clear effect of attention in behavioral results, the SSVEP results did not show an effect of hand proximity attention for the right-handers. Similarly, the SSVEP results showed a top–down attention effect only for the right-handers, although there is no particular reason to believe that left-handers did not show any effects of top–down attention. These differences between the left- and right-handers were surprising, and we will examine the issue further in Section 4.

The SSVEP amplitude revealed that the left-handers differed from the right-handers. The side where the hand was located showed a larger SSVEP amplitude for the left-handers (Fig. 9, left), whereas no such effect was found for the right-handers (Fig. 9, right). This contrasts with the effect of top–down attention, where a clear effect was shown for the right-handers (Fig. 8, right) but not for the left-handers (Fig. 8, left). This may suggest that hand proximity attention in left-handers is controlled by top–down attention. We expect a larger SSVEP amplitude at the flash location because the participants were asked to focus their attention on that location in order to perform the task. However, there were no significant differences in SSVEP amplitude between the attended and unattended locations (Fig. 8 left). One possible interpretation of the hand proximity attention of the left-handers may be the influence of top–down attention, which is attracted to the hand location instead of the flash location for an unknown reason. To further investigate this point, we analyzed the results separately for participants with and without significant hand proximity attention in the behavioral results.

**Fig. 10:**
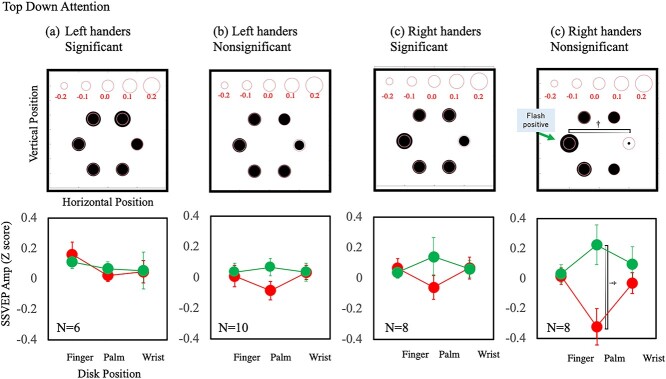
Estimation of the top–down attention effect separately for the participants with and without statistically significant effects. a) Results of left-handers with statistically significant effects. b) Results of left-handers without statistically significant effects. c) Results of right-handers with statistically significant effects. d) Results of right-handers without statistically significant effects. The notations are the same as in Fig. 8.

**Fig. 11:**
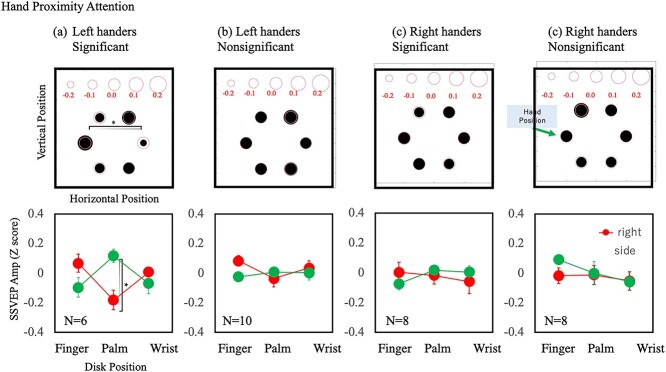
Estimation of the hand proximity attention effect separately for the participants with and without statistically significant effects. The notations are the same as in Fig. 10.

Figure 10 shows the effect of top–down attention and Fig. 11 shows the effect of hand proximity attention separately for the participants with and without significant hand proximity attention. The top–down attention effect is marginally significant at the palm location only for the right-handers without significant hand proximity attention [*F*(1,7) = 2.34, *P* = 0.052, Cohen’s *d* = 1.53]. In contrast, the effect of hand proximity attention at the palm location is the largest and was significant for the left-handers with significant hand proximity attention [*F*(1,5) = 3.87 *P* = 0.012, Cohen’s *d* = 2.14]. These results support the notion that the underlying mechanisms for hand proximity attention differ between left- and right-handers.

Thus far, the behavioral data have shown that hand proximity attention is seen even without the visual cues of hand locations, and the EEG results suggest that the effects differ between left- and right-handers. One critical question raised by these findings is the relationship between hand proximity attention and top–down attention. With or without intention, the participant’s top–down attention may direct the area around the dominant hand, and an attentional effect may be seen near the hand. This is unlikely to have happened under the conditions of the present experiment. We instructed participants before each session to focus on the location where the flashes would always be presented. Given that the information of the flash location was 100% valid, top–down attention should orient to the flash location that the participants were informed of in advance. Orienting the top–down attention near the hand was generally disadvantageous for performing a task in the present experiment. However, the SSVEP results suggest that top–down attention was oriented around the hand for the left-handers. For the right-handers, there was no SSVEP data that might explain the hand proximity attention shown by FLE measurements, whereas there were SSVEP responses that corresponded to top–down attention, suggesting different attention mechanisms for each both types of attention.

## Experiment 2: reaction time measurements

We used the FLE to measure attentional modulation in experiment 1. Although there is no specific reason to suspect that FLE might provide different results from conventional methods such as simple reaction time, we confirmed in experiment 2 that reaction time measurements showed similar effects of hand proximity attention to that measured with FLE. The participants were asked to respond to the flash presentation (reaction time task) as well as reproduce the apparent location of the moving bar relative to the flash (FLE task). They were instructed to respond as quickly as possible when flashes were presented and to memorize the location of the bar relative to the flash, so they could report it at the end of the trial. Because of the additional task involving reaction time measurements, the task in experiment 2 was more difficult than that in experiment 1.

**Fig. 12:**
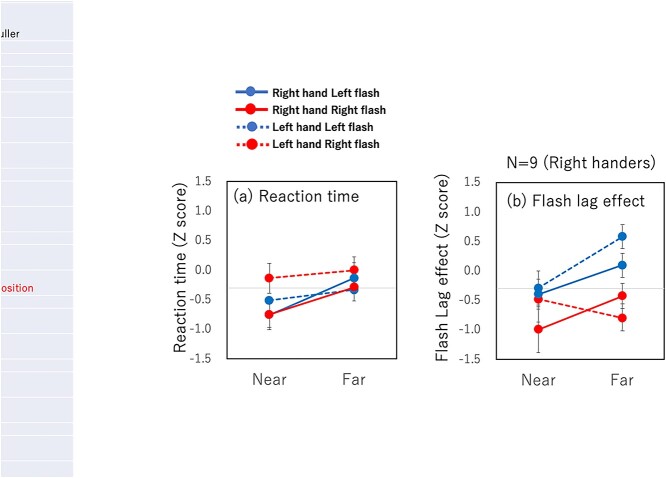
Results of experiment 2. *Z*-scores of reaction time a) and FLE b). The notations are the same as in Fig. 3.

### Method

Participants used a foot pedal to respond to the flash presentation, allowing us to measure the reaction time of stimulus detection, but we did not perform EEG measurements in this experiment. The stimuli and conditions were the same as in experiment 1. Nine right-handers participated in the experiment. The participants were instructed to press the foot pedal with their right foot to report the detection of the flash. The reaction time was the primary task, and the participant was also asked to report the perceived location of the flash after the stimulus presentation. The stimulus presentation was shortened to 1,800 ms and the flash was presented at a time randomly selected between 514 and 1,028 ms after the start of the bar rotation. Each session consisted of 36 trials. The bar and flashes were aligned in 30 trials, and the angle between them was randomly chosen between 10° and 30° in 6 dummy trials. A short brake was held after the first 18 trials in each session. The rotation direction was always clockwise. As in experiment 1, 4 combinations of hand and hand locations were used for each of the left- and right-flash locations. Each participant performed 4 sessions for each condition.

### Results

Reaction time differed between the near and far conditions (Fig. 12a), similar to the FLEs in this experiment (Fig. 12b) as well as those in experiment 1 (Fig. 3). Three-way ANOVA [flash location (right/left), hand (right/left), and distance from the hand (near/far)] showed significant main effects of distance from the hand [*F*(1,8) = 23.34, *P* = 0.001, η_p_^2^ = 0.74], with no other significant main effect or interaction. The FLE measured in the same experiment also showed a clear effect of hand proximity attention (Fig. 12b). The same 3-way ANOVA showed significant main effects of distance from the hand [*F*(1,8) = 5.79 *P* = 0.04, η_p_^2^ = 0.76]. There were significant interactions between flash location and hand [*F*(1,8) = 15.63 *P* = 0.004, η_p_^2^ = 0.5] and flash location and distance [*F*(1,8) = 7.76, *P* = 0.02, η_p_^2^ = 0.76]. No other significant main effect or interaction was observed.

The effect of hand proximity attention was found in a similar manner in both reaction time and FLE. This justifies the use of FLE for measuring the attention effect, as previously suggested (Baldo and Klein 1995; Baldo et al. 2002; Namba and Baldo 2004; Kashiwase et al. 2012, 2013; Hogendoorn 2020). The FLE results appear less reliable in experiment 2 than in experiment 1. One of the 4 conditions (right flash with the left hand) did not show an effect of hand proximity attention (red dashed line in Fig. 12b) in experiment 2, whereas all 4 conditions showed a near-hand effect in experiment 1. This can be attributed to fewer participant and trials as well as the effect of differences in the difficulty of the task.

One difference in particular may offer a clue for understanding the difference in underlying mechanisms for reaction time and FLE measurements. The FLE results showed a significant main effect of flash location in both experiments, whereas no such effect was found from the reaction time measurements. One possible reason for this difference between the left and right flashes in FLE might be differences in spatial analyses between the left and right hemispheres. Visual spatial processing (e.g. distance judgments) in the right hemisphere is believed to be superior to that in the left hemisphere (Kosslyn et al. 1989; Peters and Servos 1989; Laeng and Peters 1995). However, a simple prediction of the hemisphere difference is that stimuli presented in the left visual field, which is processed primarily in the right hemisphere, would be processed better there. The facilitation for FLE in terms of reducing the effect for the right flash found in the present experiments showed the opposite effect of the prediction. Also, we do not know why the effect was found for FLE but not for reaction time. There might be unknown differences between the hemispheres that are responsible for the smaller FLE observed for the right flash compared with the left, but we leave this question for future studies.

## Discussion

We examined whether visual processing could be facilitated around a hand under conditions in which top–down attention is controlled at a fixed location, no visual information of the hand is given, and no action is required during the stimulus presentation. First, we summarize the results of the right-handers. The FLE was found to be smaller when the stimulus was near the hand compared with when it was far from the hand, and we concluded that there is attentional modulation near the hands (hand proximity attention). Because the top–down attention was controlled at a fixed location, the attention effect near the hand is in addition to the top–down attention. Given that there was no visual information of the hand, we conclude that proprioceptive signals are the source of hand proximity attention in the experiment. Because no hand action was required during the stimulus presentation, the action of either the hand of interest or the other hand cannot be the cause of the effect. Furthermore, because hand proximity attention was evaluated by comparing conditions with a hand on a visual stimulus and with a hand far from the visual stimulus but still within the peripersonal space, we conclude that hand proximity attention can be attributed to the distance effect between the visual target and the hand. We confirmed that there is an attentional process related to hand location that is processed through the proprioceptive pathway.

The SSVEP analysis showed little or no effect of hand proximity attention, whereas the SSVEP amplitude was larger for the side of the flash presentation compared with the other side of fixation, showing the effect of top–down attention (Figs 8 and 9). This is consistent with the assumption of independent attention processes for top–down attention and hand proximity attention. If we assume that the SSVEP for luminance flickers reflects the early stages of visual processing (Shioiri et al. 2016), a lack of attentional modulation on SSVEP indicates that the process of hand proximity attention is not at an early stage, but rather a later stage. This is consistent with the general belief that the interaction between vision and proprioception occurs at later stages (Desmurget et al. 1999; Ogawa et al. 2007). The present finding of no difference in SSVEP between the near and far conditions is also consistent with previous findings. For example, Reed et al. (2013) found P3 components of the EEG signal that showed hand proximity attention for task-related stimuli but not the early EEG components. SSVEP for luminance flickers reflects mainly the early stages of visual processing, and thus no effect of hand information is expected. They also showed that hand proximity attention appears to bias attention for task-related or -unrelated stimuli in the peripersonal space. If hand proximity attention is similarly effective for any location within the peripersonal space, no difference would be expected in the SSVEP results between the near and far conditions within the peripersonal space in the present experiments.

Next, we summarize the results of the left-handers, which are rather complicated and puzzling. No statistically significant effect of hand proximity attention was found for the left-handed group. This might suggest that hand proximity attention is related to the dominant usage of the right hand, which is specific to right-handers. This is also suggested by a positive correlation between the effect of hand proximity attention and handedness score (Fig. 5). However, our analysis of the individual results suggests something different. Of the 16 left-handers, 6 showed a significant effect of hand proximity attention. This number is comparable to the 8 out of 16 right-handers who showed a significant effect of hand proximity attention. Although the left-handed group was influenced much less by hand proximity attention compared with the right-handed group, a considerable number of left-handers showed a clear effect of hand proximity attention. The SSVEP results showed interesting differences between the left-handers and right-handers. Despite the clear effect of attention in the behavioral results, the SSVEP amplitude did not show an effect of hand proximity attention but did show a top-down attention effect for the right-handers. The results for the left-handers were the opposite. The SSVEP amplitude showed an effect of hand proximity attention but not top–down attention effect (Figs 8 and 9). Separate analyses for participants with and without significant effects of hand proximity attention revealed that the difference came mostly from the left-handers with significant effects of hand proximity attention as evaluated by FLE measurements. The SSVEP amplitude of these left-handers showed a clear effect of hand proximity attention but no top–down attention effect (Figs 10 and 11). The rest of the participants showed no sign of an effect of hand proximity attention, suggesting that the hand proximity attention found for the left-handers differed from that found for the right-handers. Differences in hand proximity attention between the left- and right-handers (i.e. that it is weaker for left-handers than for right-handers) have been reported previously (Le Bigot and Grosjean 2012). Different effects between the left and right hand as well as the left and right stimuli for right-handers have also been reported (Lloyd et al. 2010; Tseng and Bridgeman 2011). Similarly, our results showed a strong hand proximity attention for the right hand of the right-handers, and less of a difference between the 2 hands of left-handers compared with right-handers. Based on these results, we consider that the process of hand proximity attention for left-handers differs from that for right-handers.

The differences between left- and right-handers in terms of hand proximity attention are possibly important for understanding the cause of handedness variation. Although the cause of handedness variation remains unclear (McManus 2019), functions potentially related to handedness have been investigated (Badzakova-Trajkov, Haberling, and Corballis 2010a; Badzakova-Trajkov, Haberling, Roberts, et al. 2010b; Willems et al. 2014). It is well known that language-related functions are lateralized for the left hemisphere (Pujol et al. 1999; Knecht et al. 2000), and most people are right-cerebrally dominant for certain nonverbal functions, including spatial attention (Cai et al. 2013) (Mesulam 1981; Fink et al. 2000; Fink et al. 2001) and the processing of faces (Yovel et al. 2008; Meng et al. 2012). It has also been shown that there is no clear relationship between handedness and laterality of right-hemisphere dominant functions, although there is a tendency of the laterality of language processing to be weaker with left-handed people (Badzakova-Trajkov, Haberling, and Corballis 2010a; Badzakova-Trajkov, Haberling, Roberts, et al. 2010b; Willems et al. 2014). Interestingly, there is a difference between left- and right-hemisphere dominant functions in terms of individual variability. For language laterality, right-handers are more consistent than left-handers, but no such strong bias is seen for right-hemisphere dominant functions, including attention-related spatial processing. This suggests that handedness is not directly related to the laterality of the attention process. The laterality of the attentional effect is typically investigated by using a bisection task and no detailed investigation has been conducted of hand proximity attention. Further investigation of the differences in hand proximity attention and top–down attention between left- and right-handers might contribute to understanding handedness variation in future.

We define the facilitation of visual processing at the location of the hand as hand proximity attention, which can be explained either by attention via the recruitment of bimodal visual–tactile neurons (Colby and Duhamel 1991; Graziano and Gross 1993; Reed, Grubb, et al. 2006a) or by preferential processing by the magnocellular visual pathway (M-pathway) (Gozli et al. 2012, 2014; Tseng et al. 2012; Goodhew et al. 2014; Huffman et al. 2015; Taylor et al. 2015). Both interpretations can be considered as an effect of attention if we define attention as the selective enhancement of activity in one or both of the following neural mechanisms: selection by visuo-tactile neurons, where the hand near a visual stimulus selectively increases the response of the neurons to the stimulus; and selection via the M-pathway, where the hand near a visual stimulus selectively increases the response of the neurons in the M-pathway. These 2 interpretations assume different underlying mechanisms, and we discuss the relationship with the results of this study.

The results for right-handers, who showed no SSVEP responses related to the hand proximity attention, support the bimodal visual–tactile neuron theory rather than M-pathway theory. Because the separation of the magnocellular and parvocellular pathways (M- and P-pathways, respectively) starts at the retina (Livingstone and Hubel 1988) and is likely to be less clear at later stages due to a variety of interactions between them (Merigan and Maunsell 1993; Takano et al. 2020), the theory predicts the neural responses (the SSVEP amplitudes in the present experiment) corresponding to hand proximity attention at the early stage of vision. However, we found no effect of hand proximity on SSVEP amplitudes for right-handers. Although separate processes originated to the M- and P-pathways are also found in much later stage of visual processing (Mishkin et al. 1983; Goodale and Milner 1992), we speculate that the effect is more clearly observable at the early stage because of interactions between the 2 pathways in a variety of visual processes (Merigan and Maunsell 1993). The bimodal visual–tactile neurons theory does not predict neural differences at the early stage of vision because visual–tactile neurons are found in brain areas far from the early visual cortices (Graziano and Gross 1993). Therefore, the neural responses that correspond to hand proximity attention would be expected at later stages of visual processing. Based on the above, we consider that the present results of no SSVEP responses related to hand proximity attention in right-handers support the bimodal visual–tactile neurons theory rather than M-pathway theory.

There are several lines of experiments that provide support for the M-pathways theory. Representative differences between M- and P-pathways include spatial and temporal resolutions, and it has been reported that performance was better on temporal-gap detection and worse on spatial-gap detection when stimuli were presented near the hands (Gozli et al. 2012; Goodhew et al. 2014). Furthermore, there are reports that object-change detection is influenced by position information but not by color information (Goodhew et al. 2014) and that visual feature binding is reduced in the near-hand space (Gozli et al. 2014). These findings are consistent with the prediction based on enhancement of the M-pathway. Reports of the effect of hand poses, including modulations of attentional prioritization by grasp postures (Reed et al. 2010; Davoli and Brockmole 2012; Thomas 2013, 2015), are also consistent with the M-pathway theory, assuming that action-related neural processes are included in the M-pathway. However, the present results may not be consistent with the M-pathway theory. We used FLE to measure the hand proximity effect. FLE becomes smaller with smaller M-pathway responses (Chappell and Mullen 2010; Hubbard 2014), meaning that the hand proximity effect should be reflected as an increase in FLE when M-pathway responses are enhanced near the hand. This does not disprove the M-pathway theory because it is possible that multiple factors contribute to the hand proximity effect. However, it suggests that the hand proximity effect shown in the present experiments is not based on the theoretical mechanism of M-pathway enhancement.

Based on the close link between action and attention, attention has been characterized as the intention of action in the “premotor theory of attention” (Rizzolatti, Riggio, et al. 1987b; Sheliga et al. 1994; Messinger et al. 2021). Hand proximity attention could also be related to action. Visually focusing one’s attention on the area around a hand in action is usually required for better control of the hand. Hand proximity attention can be the function for the purpose, working implicitly. Attention focused on the goal of hand movements has been reported, in addition to that focused on the goal of saccadic eye movements (Deubel and Schneider 1996; Deubel et al. 1998; Moore and Fallah 2004). The present study used a stationary hand at a visual stimulus and there was no action required during the stimulus presentation. However, this does not indicate that there was no action at all in the experiment. The participant was asked to use mouse buttons to report the angle of the rotating bar at the time of the flash presentation. Although the response was made after the stimulus presentation, this action might have activated the neural system sensitive to proprioceptive signals and the information of the hand position might have been updated at that time. In this sense, the location of the hand in the present experiment was the location of the manipulated object, and the hand proximity attention found might have been the same type of attention for the goal of the hand movements. Accordingly, this is not inconsistent with the premotor theory of attention, although hand proximity attention may or may not be classifiable as a type of intention.
